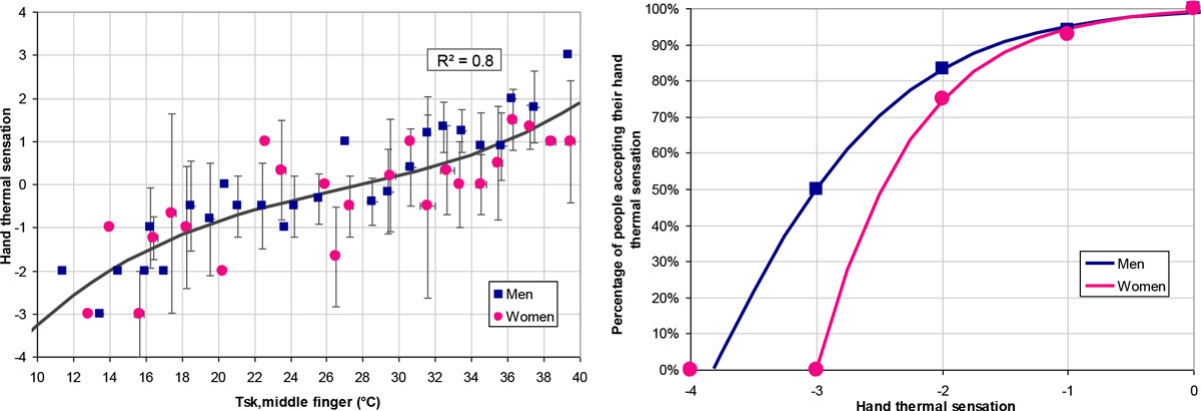# Perception of cold hands: comparison between women and men

**DOI:** 10.1186/2046-7648-4-S1-A80

**Published:** 2015-09-14

**Authors:** Thomas Voelcker

**Affiliations:** 1 Thermal Comfort Laboratory, Decathlon Sports Lab, Decathlon, Villeneuve d'Ascq, France

## Introduction

Exposure to cold generally causes hand cooling associated with a cold sensation. Literature gives various thresholds for hand skin temperature under which cold sensation is not accepted anymore: 10 °C for Hellström [1], between 15 °C and 20 °C for Havenith [2], around 21 °C for Candas [3]. In the present study, correlation between finger skin temperature and cold sensation of the hand was investigated for women and men. A relationship was also established with the percentage of people accepting this sensation in order to compare and explain how women and men differ in their perception of cold hand.

## Methods

Twenty-three subjects (9 women and 14 men) with no peripheral vascular disease walked on a treadmill for 45 minutes in a climatic chamber. Six hiking gloves were tested in four different conditions: 5 km.h^-1 ^walk with a 10 % slope at -20 °C, 5 km.h^-1 ^walk with a 10 % slope at -5 °C, 5 km.h^-1 ^flat walk at -5 °C and 5 km.h^-1 ^flat walk at 5 °C. Middle finger skin temperature (Tsk) was monitored continuously with thermocouples. Participants were asked to rate their hand thermal sensation (HTS) using a 9 points scale (from -4 "extremely cold" to +4 "extremely hot") and to determine whether this sensation was acceptable or not five times during the protocol.

## Results

The same relation between Tsk and HTS was found for women and men, with a neutral sensation (0) at 28 °C, cool (-1) at 19 °C and cold (-2) at 14 °C. However, acceptability differed between both sexes.

## Discussion

While the same percentage of women and men felt their hands comfortable for neutral (100 %) and cool (95 %) sensation, differences appeared for harsher conditions, with women showing less tolerance to cold. None of the women accepted a very cold sensation (-3) while half of the men considered it was still acceptable. For sports and leisure applications, we picked Tsk = 23 °C as the discomfort threshold for cold - both for men and women - corresponding to the point keeping 95 % of the participants with comfortable hands.

## Conclusion

This study points out the difference between thermal sensation and thermal acceptability. In cold conditions, women and men will feel the same sensation on their hands. But, women tend to be less tolerant to cold than men, especially when finger skin temperature drops under 23 °C.

**Figure 1:**